# Case of Aphonia, Successfully Treated

**Published:** 1823-08

**Authors:** Miles Partington


### Art. II.—*Case of Aphonia, successfully treated.*

By Miles Partington, Esq.

The following account of a galvanic experiment on a *dumb boy* having been inserted in several newspapers, unknown to me at the time, I am induced, by the advice of several medical friends, to attest the truth and correctness of the detail, as far as respects my knowledge of the circumstances attending the event of his recovery; and, having made the strictest inquiry of those immediately connected with Christ's Hospital, I have every reason to believe the following detail to be strictly true.

Eight months ago, a youth, about twelve years of age, named Oldham, in Christ's Hospital, went to bed at the usual hour, and in the morning rose totally dumb! He preserved every other faculty, but was obliged to write on a slate for every thing he wanted that he could not explain by signs. Every means of internal remedy, and also electricity, were resorted to without effect. Galvanism was also attempted, but was so much resisted by the boy's fears, that it could not then be applied. His general health was invariably good. At length, by strong recommendations, his fears of galvanism were overcome, and it was applied five different days. On Friday week, being the evening of the fifth application, exactly eight months (to a day), he retired to bed as usual, and awoke suddenly about eleven o'clock, making so much noise as to awaken some of his school-fellows. Their astonishment induced so much alarm, that the nurse opened the door of her adjoining apartment to learn the cause, when many voices exclaimed, "Oh! nurse, Oldham can speak again!" The nurse, doubting the fact, immediately went to him, and discovered the reality of this extraordinary phenomenon. In the morning, the boy had quite recovered his speech; and, on being asked if he felt any peculiar sensation, merely said he thought he was being galvanised, as he felt the tip of his tongue affected, together with a rumbling in his inside. His speech has continued perfect ever since.

In addition to the above statement, it may be proper to say that, some time previous to the commencement of the experiment, he was brought to my house; but, having been somewhere electrified, the boy was so much frightened on seeing a large apparatus in the room, that, considering the agitation he then laboured under, I did not think it prudent to urge him further, and he departed without being galvanised. About two or three months after, he came again, attended by a medical assistant, with a note from Mr. Field, the respectable apothecary to the hospital, assuring me that the boy was willing to submit to the experiment, and to be repeated according to my direction; and, in truth, he suffered me to proceed in a willing manner. I began with a small galvanic trough; plates, in breadth and depth, one inch; with diluted muriatic acid. Having placed a piece of insulated platina on his tongue, which, holding in his own hand, he could shift according to instruction, while I applied another conductor to different parts of the larynx, varying the direction according as I perceived the muscles to be most easily put in motion, and the vocal nerves apparently excited. By the account he gave after his recovery, a sensation of warmth always continued for some time as he returned home, and there constantly occurred an increased flow of saliva during the operation.

I am not aware that any further particulars are necessary to be stated, as every person conversant with the medical application of galvanism or electricity must know the necessity of attending to the present sensations as a guide, which admits of variation according to the state or temperament of the sensory nerves at the time of application. I deem it only necessary to add, that my young patient attended three days in the week; and it was on the morning after the fifth time that I received a grateful letter from the father, informing me of his son's entire restoration of speech, at eleven o'clock on the preceding night, having been galvanised at three o'clock on the same day, being the fifth time of attendance: and I was much gratified, a few hours after, with a visit from the boy, attended by his father; the son himself giving me, with a clear voice, the whole of the circumstances stated in the Times Newspaper, and, as I am told, copied afterwards into other papers.

P. S.—It may be proper to state, that the boy continues well at the present time.

*Orchard-street, Portman-square; June* 19, 1823.

---

Art. III.—*On Renal Inflammation.* By Dr. Kinglake.

